# Coordination‐Cage‐Catalysed Hydrolysis of Organophosphates: Cavity‐ or Surface‐Based?

**DOI:** 10.1002/chem.201904708

**Published:** 2020-02-06

**Authors:** Christopher G. P. Taylor, Alexander J. Metherell, Stephen P. Argent, Fatma M. Ashour, Nicholas H. Williams, Michael D. Ward

**Affiliations:** ^1^ Department of Chemistry University of Warwick Coventry CV4 7AL UK; ^2^ Department of Chemistry University of Sheffield Sheffield S3 7HF UK

**Keywords:** catalysis, cage compounds, host–guest chemistry, phospho-esters, supramolecular chemistry

## Abstract

The hydrophobic central cavity of a water‐soluble M_8_L_12_ cubic coordination cage can accommodate a range of phospho‐diester and phospho‐triester guests such as the insecticide “dichlorvos” (2,2‐dichlorovinyl dimethyl phosphate) and the chemical warfare agent analogue di(isopropyl) chlorophosphate. The accumulation of hydroxide ions around the cationic cage surface due to ion‐pairing in solution generates a high local pH around the cage, resulting in catalysed hydrolysis of the phospho‐triester guests. A series of control experiments unexpectedly demonstrates that—in marked contrast to previous cases—it is not necessary for the phospho‐triester substrates to be bound inside the cavity for catalysed hydrolysis to occur. This suggests that catalysis can occur on the exterior surface of the cage as well as the interior surface, with the exterior‐binding catalysis pathway dominating here because of the small binding constants for these phospho‐triester substrates in the cage cavity. These observations suggest that cationic but hydrophobic surfaces could act as quite general catalysts in water by bringing substrates into contact with the surface (via the hydrophobic effect) where there is also a high local concentration of anions (due to ion pairing/electrostatic effects).

## Graphical abstract

**Magic 8‐ball**: An octanuclear cubic coordination cage host binds organophosphate guests in the central cavity as the crystal structure of the complex with the insecticide ‘dichlorvos’ shows; but catalysed hydrolysis in aqueous solution occurs primarily at the external surface and not in the cavity.

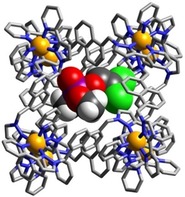

## Introduction

The cavities of self‐assembled molecular container molecules provide a fertile environment for the study of catalysis in confined spaces.^1^, ^2^, ^3^, ^4^, ^5^, ^6^, ^7^ The relatively rigid, hydrophobic cavities arise from the self‐assembly process of relatively simple metal and ligand components into hollow, pseudo‐spherical arrays and show some similarities to the binding pockets of enzyme active sites. As the size and shape of these cavities can be predicted to some extent, it is possible to design a cavity of known dimensions which will accommodate complementary guests.

The size and shape of the cavity not only determines which guests can bind but can also affect catalysis of their reactions. Raymond and Bergman have demonstrated remarkable examples of unimolecular cyclisation reactions that are catalysed because the dimensions of the host cavity are ideal for stabilisation of folded transition states or intermediates.^3^ In other cases, the combination of two substrates binding in one cavity accelerates their reaction based on the higher effective concentration they experience relative to their bulk concentrations in solution.^4^ Much of the work on catalysis of reactions in synthetic cavities has therefore focussed on the structural aspects of the containers.

**Figure 1:**
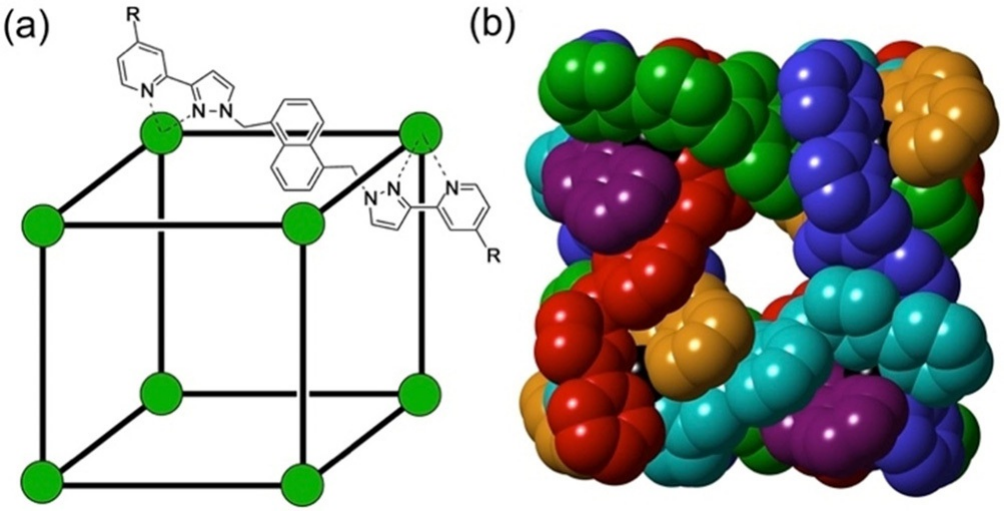
The host cage [Co_8_L_12_]^16+^, abbreviated as **H** (R=H, used in this paper as its chloride salt) and **H^w^** (R=CH_2_OH, used in related work, for example, ref. 5). (a) A sketch emphasising the cubic array of Co^II^ ions and the disposition of a bridging ligand; (b) a view of the complex cation of **H** from a crystal structure with each ligand coloured separately for clarity, emphasising the entwined ligand set and the windows in the centre of each face.

A less obvious contribution to catalysis that we have recently exploited is an electrostatic effect that can arise from charged containers when the catalysed reaction involves ions. We recently reported an example of the highly efficient (2×10^5^‐fold rate acceleration) catalysis of the Kemp elimination (base‐promoted reaction of benzisoxazole to form 2‐cyanophenolate) in the cavity of an octanuclear, approximately cubic, coordination cage **H^w^** (Figure 1) which has a charge of 16+.^5^ The catalysis was attributed to the accumulation of hydroxide ions from aqueous solution around the highly positive surface of the cage, such that the bound benzisoxazole experiences a very high local concentration of base even when the bulk pH is relatively low. This ion‐pairing effect also allows other anionic bases (phenolates) to participate in the cage‐catalysed Kemp elimination as they are less well solvated than hydroxide ions and so preferentially accumulate around the cage surface.^6^ Related examples come from Raymond and Bergman using a tetrahedral cage with tris‐catecholate vertices that has a 12− charge: the high negative charge on the host facilitated protonation of bound guests, such that acid‐catalysed reactions can occur in the cage cavity even under basic conditions.^7^

This electronic contribution to the catalysis of reactions in cage cavities has received less attention than the more obvious factors associated with recognition and binding of specific guests but is of potentially equal significance. It is similar to the effect which operates when reactions are catalysed in ionic micelles by accumulation of counter‐ions in the Stern layer around the charged surface.^8^ The cage‐catalysed reaction that we described of cavity‐bound benzisoxazole with surface‐bound basic anions^5^, ^6^ is potentially general, as it relies on two orthogonal interactions: hydrophobic binding of the substrate in the cavity,^9^ and accumulation of the anionic reaction partners (hydroxide^5^ or a phenolate^6^) around the cationic cage surface by ion‐pairing. In principle, any hydrophobic electrophile which binds in the cavity can be brought into close contact with a high local concentration of any desired anion as a reaction partner.

In order to investigate the generality of this further we have investigated the ability of our cage host to catalyse hydrolysis of a range of different substrates: specifically, organophosphates. Given the toxicity of these substrates, which has led to their use in insecticides and their obvious relationship to some chemical warfare agents, the ability of a synthetic host to bind and catalyse their destruction is clearly of interest. Indeed there have been a few other reports of catalysed destruction of organophosphates in the cavities of a metal‐organic10a, 10b and covalent organic10c frameworks, and a tetrahedral coordination cage.^11^ We have reported recently the binding of a range of alkyl phosphonates—relatively unreactive and benign simulants of G‐series chemical warfare agents—in the cavity of one of our cages in water,^12^ and this work follows on from that but exploits the catalytic activity of the cages to destroy the more reactive phosphotriester substrates. In the course of this work we have discovered—entirely unexpectedly—that it is not necessary for the substrates to be bound inside the cavity for catalysed hydrolysis to occur, but that observable catalysis can occur at the external surface, albeit with less efficiency than occurred in the Kemp elimination reaction of a cavity‐bound substrate.^5^ The interesting implication of this is that surfaces which combine cationic and hydrophobic character have the potential to act as quite general catalysts in water by bringing substrates into contact with the surface (via the hydrophobic effect) where there is also a high local concentration of anions (due to ion pairing/electrostatic effects). If catalysed reactions can occur at the external surface in this way, the high shape/size selectivity associated with guest binding in cavity will be lost. However, the catalysis, based on bringing together two components in water using orthogonal interactions, will be potentially general and could be used for a very wide range of substrate/anion combinations.

## Results and Discussion

### Choice of substrates

**Scheme 1:**
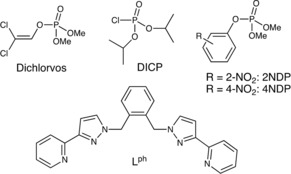
Top: the phospho‐ester guests / substrates used in this work. Bottom: the ligand L^ph^ which is used in the tetrahedral cage **T** (Figure 9).

The species that we initially investigated as possible guests (Scheme 1) were 2,2‐dichlorovinyl dimethyl phosphate (‘dichlorvos’), 2‐nitrophenyl dimethyl phosphate (2NDP) and di(isopropyl) chlorophosphate (DICP). Dichlorvos^13^ is an insecticide that has been banned in the EU since 1998 due to its toxicity—it is an acetylcholinesterase inhibitor,^14^ and is toxic to far more than just insects—but is nonetheless still in widespread use in developing countries. 2NDP is an isomer of “paraoxon‐methyl” (4‐nitrophenyl dimethyl phosphate) (4NDP) which is the active metabolite of the insecticide parathion and is also an acetylcholinesterase inhibitor.^14^ 4NDP would be an obvious choice of substrate for this work but we initially ruled it out because molecular modelling revealed that the *p*‐nitro group on the phenyl substituent would clash with the cage interior surface and prevent binding in the cavity. This issue was alleviated by use of the *o*‐nitro substituted analogue 2NDP. Dialkyl chlorophosphates are similarly reactive and of interest as simulants for G‐series chemical warfare agents,^15^ and we selected the di(isopropyl) member of the series, di(isopropyl) chlorophosphate (DICP), for investigation: compared to the methyl or ethyl analogues the higher hydrophobic surface area of the isopropyl groups should afford stronger binding in the cage cavity in water.^9^

### Solution studies of guest binding and cage/guest crystal structures

The host cage **H** is shown in Figure 1; it is the cubic M_8_L_12_ system, with a Co^II^ ion at each vertex and a bis(pyrazolyl‐pyridine) bridging ligand spanning every edge,^16^ that has been involved in our other recent work on cage‐based catalysis^5^, ^6^ and host–guest chemistry.^9^, ^17^, ^18^ The water solubility that is essential for its function is provided by the chloride counter‐ions.^6^

As a precursor to investigating any hydrolysis of the bound guests we attempted to measure their binding constants in the cage using ^1^H NMR titrations, but the fact that the guests showed clear signs of decomposition during the NMR titrations in the presence of **H**, partly due to the catalysis effect we were seeking, made this difficult. Dichlorvos proved to be in fast exchange between the free and bound states, resulting in a systematic shift in some of the paramagnetically‐shifted ^1^H NMR signals associated with the high‐spin Co^II^ cage as more dichlorvos was added (see Supporting Information, Figure S1). However, a 1:1 binding isotherm fitted the resultant data poorly due to progressive decomposition of the dichlorvos during the experiment and after several attempts we could only estimate the binding constant to be of the order of 10 m ^−1^ at 298 K. This is small compared to the best‐binding guests, which have *K* values several orders of magnitude higher,^9^, ^17^ and could arise from the hydrophilicity of the P=O group and/or the shape of the guest. Whilst the molecular volume of dichlorvos (170 Å^3^) is substantially below that required for optimal binding as predicted by Rebek's “55 % rule“,^20^ (the cage cavity has a volume of ca. 400 Å^3^), its elongated shape due to the dichlorovinyl substituent, with a bulky tetrahedral terminus, is not ideally matched to a pseudo‐spherical cavity. Similar difficulties prevented the measurement of the binding constants of the other two guests.

Accordingly we decided to estimate binding constants using the molecular docking program GOLD, for which we recently developed a custom scoring function that provides quantitative prediction of guest binding inside the cavity of **H** in water.^17^, ^18^ Analysis of the binding of our three guests using GOLD produced predicted binding constants (to two significant figures) of 31 m ^−1^ for dichlorvos, 14 m ^−1^ for 2‐nitrophenyl dimethyl phosphate and 310 m ^−1^ for DICP. Reassuringly the calculated value for dichlorvos has the same order of magnitude as the crude estimate of 10^1^ m ^−1^ that we obtained from the ^1^H NMR titrations.

**Figure 2:**
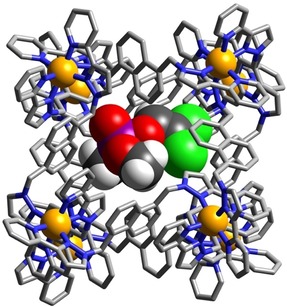
A view of the structure of **H⋅**(dichlorvos)_1.56_, with the cage **H** [in wireframe, with Co^II^ ions shown as orange spheres] containing a molecule of dichlorvos in the cavity (Co=orange, Cl=green, P=purple, O=red, C=black, N=blue) shown in one of its two disordered positions. The MeOH guest is not shown for clarity.

**Figure 3:**
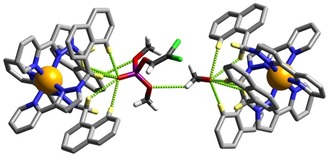
A view of the hydrogen‐bonding interactions between the dichlorvos and MeOH guests with the H‐bond donor pockets of **H** associated with the two *fac* tris‐chelate metal vertices at either end of a long diagonal of the cubic cage host. Selected H atoms are shown in yellow; the H‐bonding interactions discussed in the main text are shown as green dotted lines.

We could obtain crystal structures of the **H⋅**(dichlorvos)_1.56_ and **H**⋅DICP complexes by immersing pre‐formed crystals of **H** (as its tetrafluoroborate salt)^16^ in a methanolic solution of the appropriate guest for two hours, which resulted in uptake of the guest without loss of crystallinity, in a manner analogous to the “crystalline sponge“ method used by Fujita and co‐workers^19^ and which we have also found effective.^5^, ^6^, 9b, 9c, 18 The crystal structure of the cage/guest complex of **H⋅(**dichlorvos)_1.56_ is shown in Figures 2 and Figure 3. The crystalline sponge methodology has resulted in dichlorvos molecules being taken into the crystals of **H** in two different positions. One of the guests (Figure 2) does, as expected, lie inside the cavity, along with a molecule of methanol. The cavity is not large enough to occupy two molecules of dichlorvos, whose combined volume would be 85 % of the cavity volume. The two guests are docked into the two opposed corners of the cage associated with the *fac* tris‐chelate vertices, where a convergent set of CH protons close to a region of positive charge provided by a Co^II^ ion provides an H‐bond donor site comparable in strength to phenol.^21^ The site occupancies of the two guests are 0.36 (freely refined) for dichlorvos and 0.5 (fixed) for MeOH per asymmetric unit. The asymmetric unit is however half of the cage, which lies across an inversion centre, such that the two guests display twofold disorder between the two binding pockets, with each pocket at one end of the cage cavity therefore being occupied by a disordered mixture of 0.5 MeOH and 0.36 dichlorvos molecules, with a complete cavity therefore containing one MeOH and 0.72 dichlorvos guests overall.

Figure 2 shows the complete cage with the dichlorvos guest shown in space‐filling mode; Figure 3 shows a view of the two H‐bond donor vertices of the cage^21^ and their interactions with the dichlorvos and MeOH guests. As we have consistently observed in crystal structures of other inclusion complexes with this cage,^5^, ^6^, 9b, 9c, 18 the electron‐rich region [atom O(11G) of the P=O bond] is directed into the H‐bond donor pocket and lies 5.48 Å from Co(4). There is a set of six P=O⋅⋅⋅HC contacts with H⋅⋅⋅O separations of <3 Å, involving the convergent set of methylene CH_2_ and naphthyl CH protons in this pocket, with the shortest being 2.64 Å to H(54C) and 2.65 Å to H(61D). The bulky dichlorovinyl substituent on the P atom is directed towards one of the portals in a centre of a square face on the opposite side of the cage. The MeOH guest in the diagonally opposite *fac* tris‐chelate H‐bond donor pocket is docked via a similar network of CH⋅⋅⋅O interactions. The host cage itself has the same structure that we have reported before.^16^

**Figure 4:**
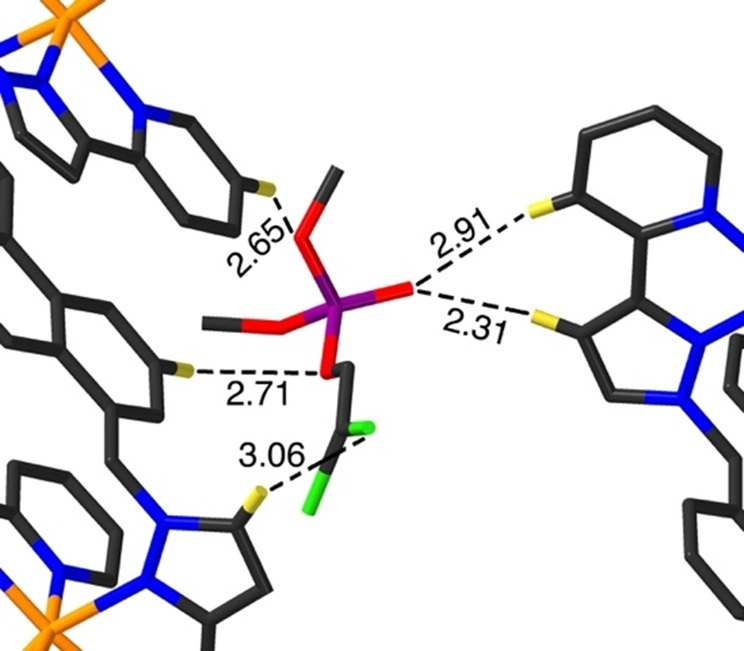
A view of the dichlorvos molecule in **H⋅**dichlorvos that is not bound in the cage cavity but lies on the outside of the cages in the space between two complex units. The range of CH⋅⋅⋅O and CH⋅⋅⋅Cl interactions between the dichlorvos molecule and the exterior surface of the cages are shown by dashed lines with separations given in Å (Cl=green, P=purple, O=red, C=black, N=blue). Selected H atoms are shown in yellow.

Figure 4 shows how the dichlorvos guest at the second site (occupancy 0.42, freely refined) lies outside the cage cavity, in the space between two cage complexes. This also shows CH⋅⋅⋅O hydrogen bonding interactions with the cage exterior surfaces; four such contacts with distances <3 Å are shown in Figure 3, with the shortest being 2.31 Å between the P=O oxygen atom O(11 H), and the externally‐directed pyrazolyl H4 proton H(44B) of an adjacent cage molecule. There is also a CH⋅⋅⋅Cl contact of 3.06 Å involving Cl(1 H).

**Figure 5:**
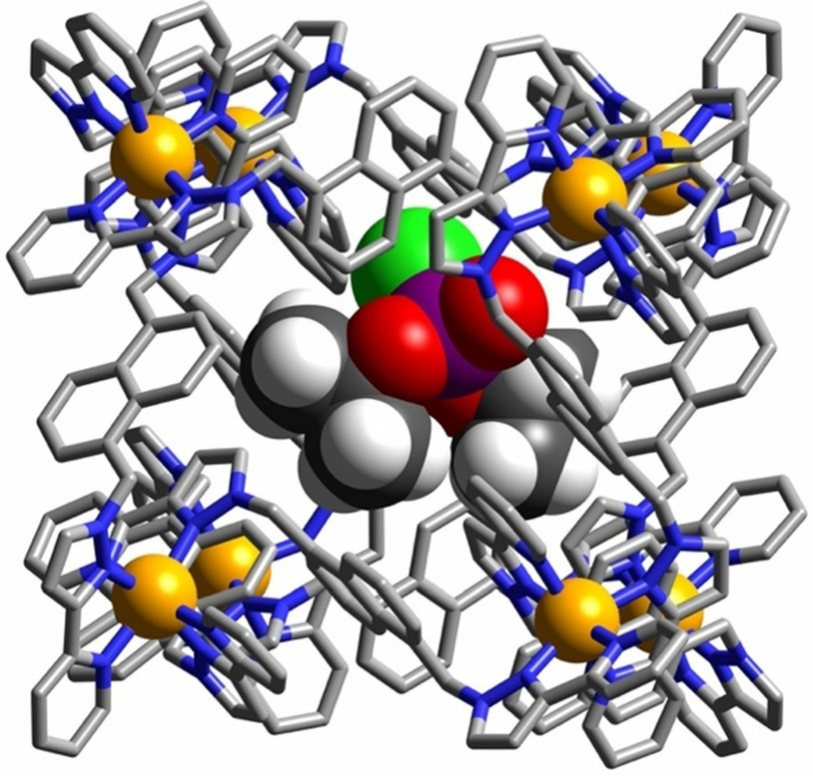
A view of the structure of **H⋅**DICP, with the cage **H** [in wireframe, with Co^II^ ions shown as small orange spheres] containing one molecule of DICP and one of MeOH in the cavity (Co=orange, Cl=green, P=purple, O=red, C=black, N=blue). The MeOH guest is not shown for clarity.

**Figure 6:**
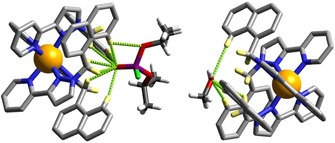
A view of the hydrogen‐bonding interactions between the DICP and MeOH guests with the H‐bond donor pockets of **H** at either end of a long diagonal of the cubic cage host. Selected H atoms are shown in yellow; the H‐bonding interactions discussed in the main text are shown as green dotted lines.

The crystal structure of **H⋅**DICP is in Figure 5 and Figure 6. The process of treating crystals of **H** with DICP has clearly generated traces of HCl from hydrolysis of DICP as several of the fluoroborate anions have been replaced by chloride in addition to the guest being taken up into the cavity (we have noted before that anion‐exchange can also be performed on single‐crystalline samples of **H** without loss of crystallinity).^6^ Although there is the usual disorder of anions, the site occupancies of the anions that could be located suggest the presence of eight tetrafluoroborate and nine chloride ions per complete cage. As only sixteen anions are required for each cage, this suggests that one of the water molecules located in the lattice is really H_3_O^+^ and the crystallographic formulation has been assigned on that basis. The important point is that one molecule of the DICP guest occupies the H‐bond donor pocket at one end of the long diagonal of the cage and one molecule of MeOH occupies the other (Figure 6), exactly as in the previous structure with dichlorvos.

The electron‐rich O atom of the P=O bond forms a similar collection of short P=O⋅⋅⋅HC contacts (there are seven such interactions in the distance range in the range 2.40–3 Å) as was seen with dichlorvos, and the MeOH guest in the opposed pocket behaves similarly. Attempts to isolate X‐ray quality crystals of **H** with the final guest studied (2NDP) were unsuccessful.

### Hydrolysis reactions

On the basis of the ion‐pairing model we proposed for the catalysed Kemp elimination,^5^, ^6^ we predicted that the high local concentration of hydroxide ions around the cage cavity at modest pH values should result in accelerated hydrolysis of dichlorvos: the expected products from this are the dimethyl phosphate anion and dichloroethanal, both of which are too small and hydrophilic to bind significantly in the cage cavity. This means that the products should readily dissociate from the cavity into the aqueous phase, affording the possibility of catalytic turnover.^5^, ^22^ The experiments were monitored using ^31^P NMR measurements in D_2_O at 298 K, buffered at pD values in the range 7.5–9.0 using borate buffer. We note that our original studies on the catalysed Kemp elimination were conducted in the presence of borate buffer; the excess of borate anions clearly does not prevent accumulation of hydroxide ions around the cationic cage surface.^5^, ^6^ A 16 mm solution of dichlorvos was monitored under these conditions, either with or without added **H** (as the chloride salt): the hydrolysis of dichlorvos was readily followed by disappearance of the signal at −3 ppm for dichlorvos and the appearance of a new signal at +3 ppm for dimethylphosphate.^23^

**Figure 7:**
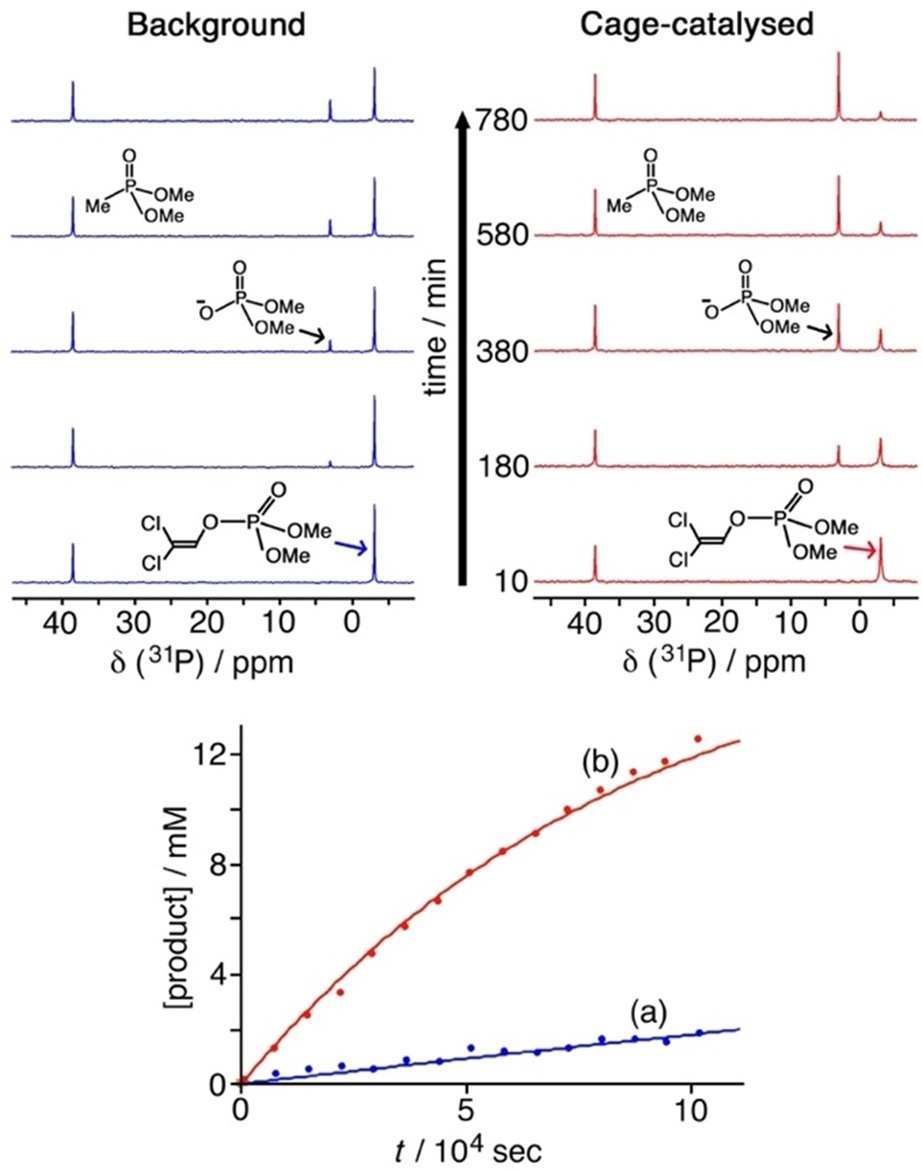
Cage‐catalysed hydrolysis of dichlorvos [16.6 mm dichlorvos in borate‐buffered D_2_O at pD 7.7 and 298 K, in the absence of cage **H** (blue traces) or the presence of **H** (0.64 mm; red traces)]. Top: evolution of ^31^P NMR spectra showing consumption of dichlorvos (−3 ppm) and appearance of dimethylphosphate (+3 ppm); a known amount of dimethyl methylphosphonate (*δ*=39 ppm) was present as a calibrant to allow quantitative integration of the ^31^P signals. Bottom: comparison of rates of product appearance in the absence and presence of **H**.

Typical results are shown in Figure 7. The observed first‐order rate constant for the hydrolysis of dichlorvos in the absence of cage **H**, *k*_uncat_, is 1.0×10^−6^ s^−1^ at pD 7.7 (298 K). Under the reaction conditions used, in the presence of 0.64 mm **H** as catalyst, the first order rate constant for appearance of product is an order of magnitude faster, at 1.4×10^−5^ s^−1^. This enhancement scales linearly with concentration of **H**, showing that the reaction is also first‐order in catalyst, and so the second‐order rate constant for the catalysed reaction *k*_2_=(1.4×10^−5^ s^−1^)/(0.64 mm)=0.02 m ^−1^ s^−1^ (Table 1, line 1).

**Table 1:** Summary of catalysed reactions rates using the range of cages and substrates discussed in this paper. For more detail see Supporting Information.

Catalyst	Conditions^[a]^	Substrate	*k*_2_ [m ^−1^ s^−1^]	*+/*− [%]^[b]^
**H** (0.64 mm)	**A**	dichlorvos (16.6 mm)	0.02	2
**H** (0.78 mm)	**A**	2NDP (15.5 mm)	0.008	3
**H** (0.21 mm)	**B**	2NDP (0.5 mm)	0.019	1
**H** (0.21 mm)	**B**	2NDP (0.5 mm)+ 62.5 mm NaCl	0.0075	1
**H** (0.95 mm)	**C**	4NDP (17.5 mm)	0.0068	2
**T** (0.95 mm)	**B**	2NDP (1 mm)	0.021	1
**T** (0.95 mm)	**B**	4NDP (1 mm)	0.015	1

[a] Conditions **A**: ^31^P NMR spectroscopy, D_2_O, pD 7.7, 298 K. Conditions **B**: UV/Vis spectroscopy with a micro‐plate reader monitoring formation of 2‐ or 4‐nitrophenolate products, H_2_O, pH 8.5, 303 K. Conditions **C**: ^31^P NMR spectroscopy, D_2_O, pD 8.5, 298 K. [b] Calculated uncertainty in *k*_2_ value arising from the fitting, rounded up to the nearest 1 %.

These observations are consistent with having a low binding constant (estimated as ca. 30 m ^−1^) for formation of the **H⋅**dichlorvos complex, as this means that most of the **H** is not bound to dichlorovos under the conditions of the experiment and that the rate of decrease in dichlorovos concentration should therefore obey the first order rate law. If we make the assumption—which was true for our previous work on the Kemp elimination^5^, ^6^ and is clearly possible as demonstrated by the structural data described above—that the catalysed reaction occurs via the cavity‐bound guest (and non‐bound guest reacts at the background rate), then by taking the fraction of bound guest into account we find that the rate constant for the cage‐catalysed reaction, *k*_cat_, would be 7×10^−4^ s^−1^: a rate acceleration *k*_cat_/*k*_uncat_ of about 700 fold for the cage‐catalysed reaction under these conditions.

Very similar behaviour was observed for catalysed hydrolysis of 2NDP, again following the reaction by ^31^P NMR spectroscopy. At a pD of 7.8 the background hydrolysis (no cage present) had an observed rate constant *k*_uncat_ of 6.0×10^−7^ s^−1^. In the presence of 0.78 mm **H** under the same conditions the rate of appearance of the product was an order of magnitude faster at 6.6×10^−6^ s^−1^, giving *k*_2_=0.008 m ^−1^ s^−1^ (Table 1, line 2) Taking into account the calculated binding constant of 14 m ^−1^ this would afford a *k*_cat_ value of 6×10^−4^ s^−1^—apparently giving a similar rate enhancement (*k*_cat_/*k*_uncat_≈1000 fold) as was observed with dichlorvos as substrate—again based on the assumption that the catalysis is occurring only on the small fraction of guest that is bound in the cage cavity. Representative reaction profiles and curve fittings to rate constants are shown in Supporting Information, Figures S2–S5.

DICP proved more difficult to analyse because its greater reactivity means that the background hydrolysis rate is already high, so any enhancement arising from cage‐based catalysis was more difficult to detect. In addition, hydrolysis of DICP generates additional chloride which—as we established recently—has a substantially inhibiting effect on the cage‐based catalysis.^6^ Accordingly this was not pursued further.

### Control experiments: Investigation of catalysis at the exterior cage surface

In order to confirm that catalysis of the hydrolysis reactions for dichlorvos and 2NDP requires the substrates to be bound inside the cage cavity, an essential control experiment is to block the cavity with an unreactive but strongly binding inhibitor. For this purpose we use cycloundecanone whose high hydrophobicity and ideal match for the cavity size afford very strong binding in water.9b In our studies on the Kemp elimination, addition of this inhibitor resulted in complete loss (within experimental error) of the catalytic rate enhancement,^5^, ^6^ with the reaction rate reverting to the uncatalysed background level.

With dichlorvos and 2DNP as substrates we were surprised to find that addition of cycloundecanone to the reaction mixture resulted in no significant change in the reaction rate, despite the cycloundecanone being bound in the cage cavity (as observed by ^1^H NMR spectroscopy which shows clear paramagnetically shifted signals between 0 and −10 ppm for bound guests).^24^ Reactions investigated in parallel with and without cycloundecanone under otherwise identical conditions showed the same reaction progress profiles within experimental error; and addition of cycloundecanone to a reaction after it was underway showed no noticeable discontinuity in the progress profile, with the reaction continuing unchanged (see Supporting Information, Figure S6). Clearly, in these cases, binding of the substrates in the internal cavity is not necessary for catalysis.

We rationalise this behaviour by assuming that the substrate can also interact with the cage via association with the cage exterior surface, which displays the same hydrophobic components as the interior cavity, and the substrate is thereby brought into the region around the cage which experiences ion pairing with hydroxide ions, providing a higher local concentration. This exterior‐binding pathway becomes the default under the experimental conditions used, in contrast to the Kemp elimination reactions where much stronger binding of the substrate benzisoxazole inside **H** meant that the cavity‐based reaction dominated and could be switched off by displacement of the guest using cycloundecanone.^5^, ^6^ Our data implies that the small fraction of internally bound substrate (ca. 1 %) does not contribute significantly to the observed hydrolysis reaction as this overall rate does not change when interior binding is excluded, and so reaction in the cavity interior is clearly less efficient than on the exterior surface. Possibly, the electrophilic phosphorus of the substrate is not positioned to allow successful attack by hydroxide through the windows in the cage sides. Given this, the *k*_cat_/*k*_uncat_ values of around 10^3^ calculated above based on the assumption that catalytic hydrolysis only takes place through the cavity‐bound species are not meaningful.

The possibility of catalytic activity at the exterior surface of the cage facilitated by weak interactions that bring cage and substrate together suggested three additional sets of experiments, as follows. For these experiments we focussed particularly on the catalysed hydrolysis of 2NDP, as the liberation of 2‐nitrophenolate is easily followed by its characteristic intense UV/Vis absorption at 420 nm which allows experiments under many different conditions to be conveniently performed in parallel by UV/Vis spectroscopy using a plate‐reader (all plate‐reader experiments were performed at 303 K due to the operating temperature inside the machine which made 298 K difficult to maintain).

**Figure 8:**
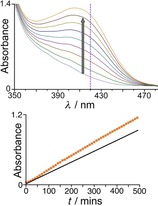
Cage‐catalysed hydrolysis of 2NDP. Top: evolution of UV spectra showing production of product 2‐nitrophenolate in the presence of cage **H**: its appearance was measured by absorbance at 420 nm (dashed line). Bottom: comparison of rates of product appearance (conditions: 0.5 mm 2NDP in borate‐buffered H_2_O at pH 8.55 and 303 K) in the absence of cage **H** (black line) or the presence of **H** (0.21 mm; orange dots).

For the next control experiment we investigated inhibition of the catalysed hydrolysis by excess chloride (Figure 8). As chloride ions are easier to desolvate than hydroxide, they bind to the cage surface more readily and thereby displace hydroxide. For the Kemp elimination on cavity‐bound benzisoxazole, addition of a large excess of chloride to the reaction completely switched off catalysis and reduced the reaction to the background rate, even though the benzisoxazole remained in the cage cavity, consistent with loss of hydroxide from the vicinity of the cage surface.^5^ In contrast, with 2NDP as substrate, addition of a large excess of chloride had only a modest inhibiting effect on the hydrolysis: for example addition of 62.5 mm NaCl to an experiment containing 0.21 mm **H** (and hence 65.9 mm chloride including the 16 counter‐ions already associated with **H**), and 0.5 mm 2NDP (at 303 K, pH 8.55) resulted in a reduction in the rate of 2‐nitrophenolate formation by only 60 % (see Table 1, entries 3 and 4, and Supporting Information, Figure S7). This is again consistent with an exterior‐surface binding model: the 2NDP will be exposed to the bulk solvent in a way that it is not when cavity‐bound, so that it can be attacked by hydroxide. The displacement of hydroxide ions bound to specific sites at the faces of the cage has a smaller effect, as other weakly associated hydroxide ions can still participate. If the substrate is in the cavity, only the anions at key locations can participate—and substitution with chloride then leads to a much more substantial loss of activity.

Finally, we investigated some cage/guest combinations in which the substrate cannot bind in the cavity. The substrate 4NDP—as noted earlier—is not expected to bind in the cage cavity of **H** according to molecular modelling. 4NDP showed a very similar enhancement of its rate of hydrolysis in the presence of **H** as did 2NDP. For example using 0.95 mm **H** and 17.5 mm 4NDP at pD 8.55 the rate constant for appearance of product is 7.8×10^−6^ s^−1^, compared to 1.3×10^−6^ s^−1^ in the absence of catalyst, giving *k*_2_=0.0068 m ^−1^ s^−1^ (Table 1, line 5; and Supporting Information, Figure S8): as this substrate does not bind in the cavity this, again, can only be an exterior surface effect.

The implication of this is that a smaller cage with a smaller cavity—incapable of binding any substrates of this size—could act as a catalyst for organophosphate hydrolysis in weakly basic solution, and so it proved in the final control experiment. The smaller tetrahedral cage [Co_4_(L^ph^)_6_](BF_4_)_8_ was reported by us some time ago^25^ and has an internal cavity only large enough to bind a tetrafluoroborate (or perchlorate) ion. This could be converted to the water‐soluble chloride salt (abbreviated hereafter as **T**, for tetrahedron) by anion exchange with Dowex resin, and an aqueous solution of **T** as its chloride salt catalysed hydrolysis of both 2NDP and 4NDP to a similar extent. Using 0.95 mm **T** and 1 mm substrate (pH 8.55, 303 K) hydrolysis of 4NDP was accelerated from 3.0×10^−6^ s^−1^ with no catalyst to 1.7×10^−5^ s^−1^ with catalyst present: and the rate constant for hydrolysis of 2NDP was accelerated from 5.0×10^−6^ s^−1^ with no catalyst to 2.5×10^−5^ s^−1^ with catalyst present. These give rate constants for the cage‐catalysed reaction of *k*_2_=0.015 and 0.021 m ^−1^ s^−1^ for cage **T** with 4NDP and 2NDP, respectively, under these conditions (Table 1, lines 6 and 7; see also Supporting Information, Figure S9). Interestingly the smaller cage **T** is a comparably good catalyst to **H** for the same substrate under the same conditions (compare lines 3/6, and 5/7, in Table 1): the smaller surface area and lower overall charge imply a similar charge density and hence ability to accumulate anions, and the surface composition is similar between the two types of cage structure (**T** and **H**) implying a similar hydrophobic character to the exterior surface.

**Figure 9:**
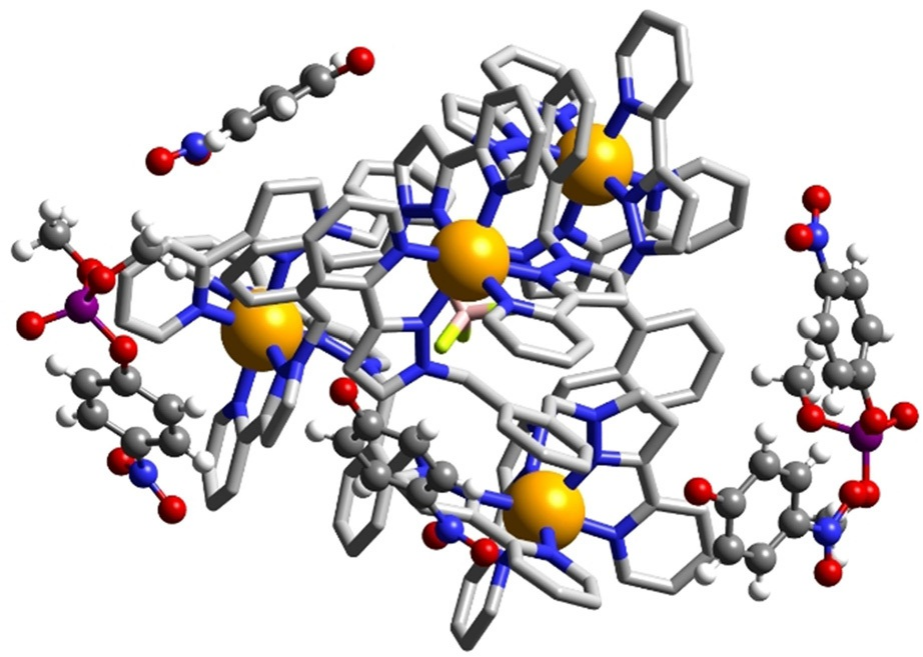
View of the structure of **T⋅**(4‐nitrophenolate)_3_⋅(4NDP)_2_ showing the presence of both starting material (4NDP) and product (4‐nitrophenolate anions) around the exterior surface of the tetrahedral cage cation [Co_4_(L^ph^)_6_]^8+^ (Co=orange, Cl=green, P=purple, O=red, C=black, N=blue).

Whilst investigating the hydrolysis of 2NDP by **T**, a solution left standing for three days in an NMR tube produced a crop of X‐ray quality crystals which proved to be the tetrahedral cage that had co‐crystallised with both 2NDP substrate and the hydrolysis product 2‐nitrophenolate (Figure 9). Whilst such a structure is not in itself an indication of the catalytically relevant species in solution, it nevertheless provides a pleasing insight into the structures available to the catalyst, starting material and product in a single assembly. The complex cage cation [Co_4_(L^ph^)_6_]^8+^ is the same edge‐bridged tetrahedral assembly that we have reported before, with four *fac* tris‐chelate metal ion vertices all having the same chirality and overall *T* molecular symmetry.^25^ The small central cavity contains a disordered mixture of a BF_4_^−^ anion and a chloride ion: the essentially complete encapsulation of the cavity by the cage—there are no portals in the faces—mean that the central, tightly bound anion^25^ has not fully exchanged for chloride. The channels between the cage cations are occupied by a mixture of nitrophenolate anions and unreacted 4NDP molecules which form a variety of contacts with the cage exterior surface. In particular the H‐bond accepting P=O oxygen atom [O(12G)] forms CH⋅⋅⋅O contacts with the CH hydrogen atoms C(35E) and C(36E) from a pyridyl ligand (the H⋅⋅⋅O separations are 2.62 and 2.99 Å respectively).

### Comparison of cavity‐based and exterior surface‐based catalysis

In our earlier work concerning the cage‐catalysed Kemp elimination,^5^ the ratio of the second‐order rate constants for catalysis by the cage and by hydroxide is 440:1. Assuming that it is hydroxide associated with the cage that is involved in the reaction, this gives a measure of the effect of the cage on the transition state relative to water (as the two reactions share a common ground state). Taking the same approach for the reaction of 2NDP with hydroxide, the second order rate constant for the background reaction in the absence of cage (measured by us) is 0.1 m ^−1^ s^−1^, which can be compared with the value of 0.008 m ^−1^ s^−1^ for the cage‐catalysed reaction (see earlier). Accordingly, the ratio of the second‐order rate constants for catalysis of 2DNP hydrolysis by the cage and by hydroxide is now 0.08: less effective by a factor of around 5000 compared to the Kemp elimination reaction. Given that the idea of a saturating rate is not applicable here as the substrate is almost entirely not in the cavity, this factor of 5000 difference between the rate acceleration we observed for the Kemp elimination^5^ and for 2DNP hydrolysis (this work), provides a way to compare the effectiveness of cavity‐catalysed and surface‐catalysed reactions under the prevailing conditions.

We need to take account of the fact that in this work we have used **H** as its chloride salt (with 16 equivalents of chloride to balance the cage charge). This in itself has a substantially inhibiting effect: we showed recently that use of the chloride salt of the cage, for synthetic convenience and good water solubility,^6^ slowed down the Kemp elimination by a factor of approximately 100 compared to the original cage‐catalysed reaction in which water solubility was provided by external substituents.^5^ Taking account of this, we can suggest that the effect of exterior surface catalysis compared to cavity‐based catalysis results in an additional reduction in the catalysed second‐order rate constant by a factor of around 50, which can reasonably be ascribed to the beneficial effect of the cavity in positioning the substrate at the centre of a shell of desolvated, surface‐bound anions that can access the reaction site of the substrate. This positioning effect can also be inhibitory, if the mode of substrate binding prevents the surface‐bound anions from reaching the required reaction site.

## Conclusions

This work leads to the clear conclusion that, whilst the coordination cage **H** can act as both (i) a host which encapsulates a range of small molecule organophosphate guests and (ii) a catalyst for their hydrolysis by surface‐bound anions, these two phenomena need not go together. The substrate/guest associates with the cage surface—interior or exterior—via the hydrophobic effect, and possibly additional polar interactions (*cf*. the crystal structures in which substrates show hydrogen‐bonding interactions with exterior cage surfaces); and—as shown in earlier work—hydroxide anions accumulate around the cage surface because of its high positive charge. It is not necessary for the substrate to be located inside the cavity, however, to be in close proximity with the high local concentration of anions. As one face of the cage surface (interior or exterior) is chemically similar to the opposite one, a catalysed reaction between substrate and anions can occur even when the cavity is blocked by a strongly binding inhibitor, or when the substrate is too large to bind inside the cage cavity. However, if the substrate does bind well in the cavity it will be fully encapsulated by the shell of anions and experience a higher local concentration of anions than a substrate in contact with the exterior surface; and therefore undergo particularly effective catalysis if the favoured orientations are suitable for reaction to occur.^5^ We note that there have been other examples of host/guest chemistry associated with coordination cages in which guests interact with the exterior surface of cages. These include tetraalkylammonium cations binding to the exohedral surface sites of an anionic tetrahedral cage;^26^ perchlorate anions occupying surface sites rather than an internal cavity site in a pentagonal‐bipyramidal assembly;^27^ and binding of tetraphenylborate anions to the exterior surface of a cubic cage acting to prevent uptake of smaller guests into the cavity via an allosteric effect.^28^

The importance of this observation is that any surface that combines the two properties of being both hydrophobic and cationic^29^, ^30^ may be able to act as a catalyst for reactions of hydrophobic organic species with anions in a similar way by co‐location of the two reaction partners using orthogonal interactions. This combination of characteristics underpins the cation‐pi interaction in water,^29^ for example, and has been suggested as contributing to molecular recognition and catalytic processes involved in the origin of life^30^ as well as molecular recognition in a range of supramolecular systems^31^ and examples of catalysis at the surface of ionic micelles.^32^ The loss of the selectivity associated with cavity binding in a cage means that this type of catalysis will be undiscriminating in terms of substrate size and shape: but the converse is a potentially broad applicability to a wide range of substrates. In particular this type of catalysis may be applicable not just to the surfaces of cationic cages but to metal/ligand nanosheets—exfoliated 2D layers derived from metal‐organic frameworks—which have been recently become of interest for their solution chemistry.^33^ Exploring the scope and generality of this catalysis will be the focus of future work.

## Experimental Section

All experimental details are in the Supporting Information; this includes details of kinetic analyses, tables of reaction rate data under a range of different conditions, and crystallographic data for the three structures.

## Conflict of interest

The authors declare no conflict of interest.

## Supporting information

As a service to our authors and readers, this journal provides supporting information supplied by the authors. Such materials are peer reviewed and may be re‐organized for online delivery, but are not copy‐edited or typeset. Technical support issues arising from supporting information (other than missing files) should be addressed to the authors.

Supplementary
