# Supplementary material for: Coordination‐Cage‐Catalysed Hydrolysis of Organophosphates: Cavity‐ or Surface‐Based?
Source: Chemistry. 2020 Feb 6;26(14):3065–73. doi: 10.1002/chem.201904708 (PMC7079011; doi:10.1002/chem.201904708)
Supplement: Supplementary file 1 — Supplementary [file CHEM-26-3065-s001.pdf]

CHEMISTRY
A European Journal

Supporting Information

Coordination-Cage-Catalysed Hydrolysis of Organophosphates: Cavity- or Surface-Based?

Christopher G. P. Taylor,[a] Alexander J. Metherell,[b] Stephen P. Argent,[a] Fatma M. Ashour,[b] Nicholas H. Williams,*[b] and Michael D. Ward*[a]

chem_201904708_sm_miscellaneous_information.pdf

Experimental details

Instrumentation used was as follows. NMR spectroscopy was performed at 298K with a Bruker AV3-400 instrument. pH measurements were performed with a Hamilton Spintrode pH combination electrode calibrated with standards at pH 7.00 and 10.01. UV/Vis spectra were recorded using an IMPLEN NanoPhotometer C40; catalysed reactions monitored by UV/Vis spectroscopy were performed using a BMG Labtech ClarioStar UV platereader at 303K. All chemicals were purchased from commercial sources and used as supplied unless otherwise stated.

Preparations of materials and solutions
Cubic cage **H**•$(BF_4)_{16}$ was prepared according to the previously published methods.[S1,S2] It was converted to the water-soluble chloride form according to the previously published procedure[S6] and the concentration of cage after ion-exchange was confirmed by the optical density of the sample at the absorption maximum of 292 nm ($\varepsilon = 3.088 \times 10^5\ M^{-1}\ cm^{-1}$).

Tetrahedral cage **T**•$(BF_4)_8$ was prepared and purified as described previously.[S7] The chloride salt **T** was prepared following the procedure for **H** and the concentration of cage confirmed by the optical density of the sample at the absorption maximum of 244 nm ($\varepsilon = 1.390 \times 10^5\ M^{-1}\ cm^{-1}$).

Buffered solutions of **H** and **T** (as chloride salts) for the catalysis studies were prepared using appropriate quantities of solid boric acid and anhydrous borax. For example, for 200 mM buffer (relative to boric acid) at pH 8.55 a 50:50 mixture of the acid and conjugate base was added which was 100 mM of boric acid and 25 mM anhydrous borax.

Predicted binding constants for the guests 2,2-dichlorovinyl-dimethyl phosphate (dichlorvos), di(isopropyl)chlorophosphate (DICP), and 2-nitrophenyl-dimethyl phosphate (2NDP) in host **H** in water were determined using the molecular docking programme GOLD with the previously published scoring function.[S3,S4] The binding constant for the ^{31}P-NMR marker, dimethyl methyl phosphonate with the cage complex, has previously been published.[S5]

	ligand clash	ligand torsion	part buried	non polar	ligand flexibility	
Weighting factors	-4.4803	0.02192	0.21624	-0.1024	-0.3599	k_{calc}
dichlorvos	0	0.2723	-4.963	-39.016	4	30.84
2NDP	0	0.464	-8.2939	-46.221	5	14.16
DICP	0	1.0301	-3.6939	-46.011	4	313.82

Table S1. Parameters used in the scoring function to determine calculated binding constants of guests using GOLD.[S3,S4]

Determination of binding constant by ^{1}H-NMR spectroscopy.
^{1}H-NMR titrations to measure the binding constant of dichlorvos in cage **H** were performed according to the previously published method.[S8] The guest was found to be in fast exchange; the graphs of δΔ vs guest concentration could be fitted approximately to a 1:1 binding model but gradual hydrolysis of the dichlorvos during the course of the measurements means this result is not accurate and therefore should only be determined as an order-of-magnitude value (10 M^{-1}).

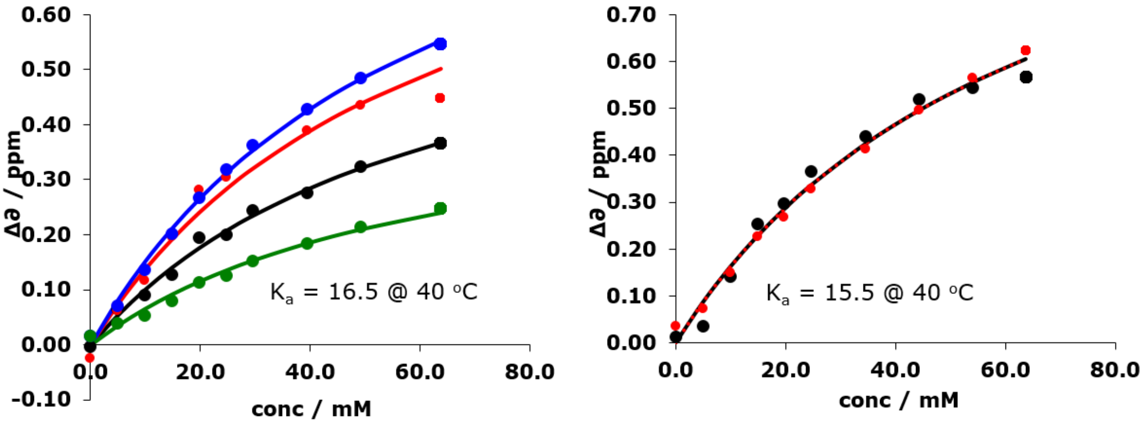

Fig. S1. Binding isotherms from ^{1}H-NMR data (@ 40°C) to determine the binding constant for **H** (0.2mM) with dichlorvos

Catalysed hydrolysis measurements monitored by ^{31}P-NMR spectroscopy.
Stock solutions of **H** in D_2O were buffered using the boric acid / borate combination as described above. For each experiment two NMR tubes were set up: 500 µL of buffered cage stock solution was added to the first and 500 µL buffer solution (no cage) was added to the second. Small amounts of NaOD (15 M) were added as required to adjust pD values. An accurate reading from the pH probe was recorded for each tube. The substrates dichlorvos or 2NDP were then added (typically 1 – 2 µL per experiment). 0.5 µL of DMMP (dimethyl-methyl phosphonate) was also added as an internal ^{31}P calibrant.

The two tubes were kept in identical conditions and ^{31}P-NMR spectra were measured for each tube in turn and repeated around every 2 hrs. Progress of the reaction, as shown by relative amounts of starting material and product, was determined by careful integration of the ^{31}P signals using deconvolution if necessary. MatLab was used to create linear fits of the ln (starting material concentration) *vs.* time to determine the first order rate constants. Data are summarised on the next page.

Substrate Name	Substrate Conc (mM)	pH	K_{bk} (s^{-1})	RMSE	% error	
Dichlorvos	16.6	7.71	1.014E-06	0.008	0.4%	
	16.6	7.75	1.168E-06	0.010	0.5%	
	16.6	7.76	1.132E-06	0.009	0.5%	
	16.3	8.28	3.115E-06	0.011	0.5%	
	16.3	8.48	3.812E-06	0.009	0.4%	
	16.3	8.48	3.656E-06	0.011	0.5%	
	16.6	8.77	6.840E-06	0.009	0.4%	
	16.6	8.79	7.031E-06	0.013	0.6%	
	16.0	8.83	6.382E-06	0.008	0.4%	
	16.0	8.97	8.502E-06	0.010	0.5%	
2NDP	15.5	7.85	5.946E-07	0.009	0.4%	
	16.1	7.90	4.990E-07	0.011	0.5%	
	16.1	7.90	4.688E-07	0.011	0.5%	
	15.8	8.30	1.010E-06	0.008	0.4%	
	15.8	8.32	1.094E-06	0.010	0.5%	a
	15.8	8.49	1.405E-06	0.010	0.5%	
	14.9	8.55	1.461E-06	0.011	0.5%	
	15.5	8.92	2.662E-06	0.136	6.5%	
	15.5	8.92	2.515E-06	0.011	0.5%	
	15.5	8.98	3.044E-06	0.010	0.5%	
4NDP	17.5	8.55	1.285E-06	0.012	0.6%	h

$K_2 = (K_{obs}-K_{bk})/[Cage]$

$$\% \text{ error} = -2 \times \frac{RMSE}{\ln([Substrate])}$$

Substrate Name	Substrate Conc (mM)	pH	K_{obs} (s^{-1})	Calc_K_{bk} (s^{-1})	Cage Name	Cage Conc (mM)	K_2 ($M^{-1}s^{-1}$)	RMSE	% error	
Dichlorvos	16.6	7.65	1.357E-05	9.565E-07	H	0.639	1.97E-02	0.034	1.6%	e
	16.6	7.65	1.582E-05	9.646E-07		0.639	2.32E-02	0.169	8.3%	
	16.6	7.68	1.556E-05	1.006E-06		0.639	2.28E-02	0.188	9.2%	
	16.3	8.25	2.823E-05	2.630E-06		0.627	4.08E-02	0.243	11.8%	
	16.6	8.74	3.821E-05	6.009E-06		0.639	5.04E-02	0.079	3.9%	
	16.6	8.75	3.928E-05	6.111E-06		0.639	5.19E-02	0.063	3.1%	
	16.0	8.76	3.409E-05	6.215E-06		0.616	4.53E-02	0.067	3.2%	
	16.0	8.84	3.964E-05	7.172E-06		0.616	5.27E-02	0.098	4.8%	
	19.9	8.05	1.656E-05			0.500		0.027	1.4%	f
	19.9	8.05	1.613E-05			0.500		0.028	1.4%	g
2NDP	16.1	7.61	5.776E-06	3.421E-07	H	0.639	8.50E-03	0.018	0.9%	
	16.1	7.63	1.445E-06	3.530E-07		0.161	6.76E-03	0.009	0.5%	
	16.1	7.76	5.739E-06	4.324E-07		0.807	6.57E-03	0.011	0.5%	
	15.5	7.79	6.636E-06	4.567E-07		0.777	7.95E-03	0.053	2.5%	d
	15.8	8.25	1.247E-05	9.292E-07		0.627	1.84E-02	0.072	3.5%	
	15.8	8.28	1.292E-05	9.814E-07		0.627	1.90E-02	0.055	2.7%	
	15.8	8.32	1.235E-05	1.037E-06		0.627	1.80E-02	0.059	2.8%	b
	14.9	8.65	9.647E-06	1.749E-06		0.750	1.05E-02	0.029	1.4%	
	15.5	8.76	4.380E-06	2.076E-06		0.123	1.87E-02	0.020	0.9%	c
	15.5	8.79	1.639E-05	2.159E-06		0.616	2.31E-02	0.047	2.3%	
	15.5	8.80	1.255E-05	2.210E-06		0.777	1.33E-02	0.035	1.7%	
	15.5	8.82	1.179E-05	2.262E-06		0.616	1.55E-02	0.029	1.4%	
4NDP	17.5	8.55	7.769E-06	*6.484E-06*		0.950	6.83E-03	0.033	1.6%	k

Table S2. Data for hydrolysis of dichlorvos and 2NDP with/without cage **H**, determined by ^{31}P-NMR spectroscopy. Highlighted rows: *a, b, c* (see **Fig. S2**); *d, e, k* (see main text, **Table 1**); *f, g* (see **Fig. S6**); *h, k* (see **Fig. S8**).

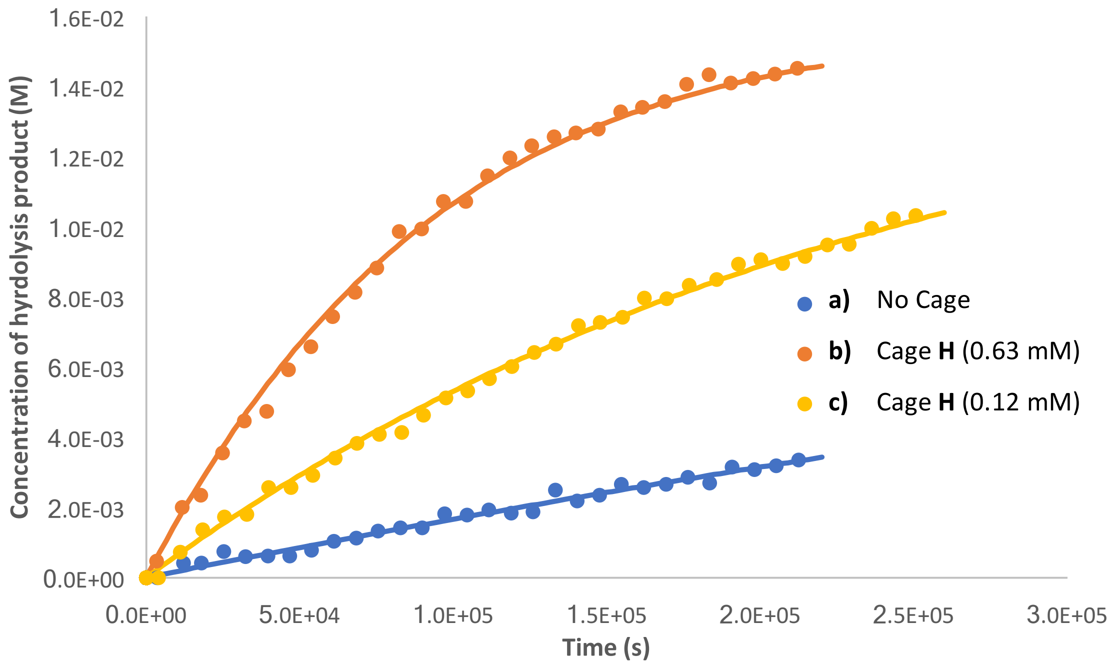

Fig. S2. Representative set of ^{31}P NMR measurements showing the accumulation of the product from the hydrolysis of 2NDP: **a)** pD 8.32, 2NDP (15.8 mM), no Cage; **b)** pD 8.32, 2NDP (15.8 mM), **H** (0.627 mM); **c)** pD 8.76, 2NDP (15.5 mM), **H** (0.123 mM); These experiments are highlighted in **Table S2** (entries labelled *a, b, c* respectively). Illustrative calculations of the rate constants for the measurements in **Fig. S2 (a)** and (**b)** are shown below in **Fig S3** and **Fig S4** respectively.

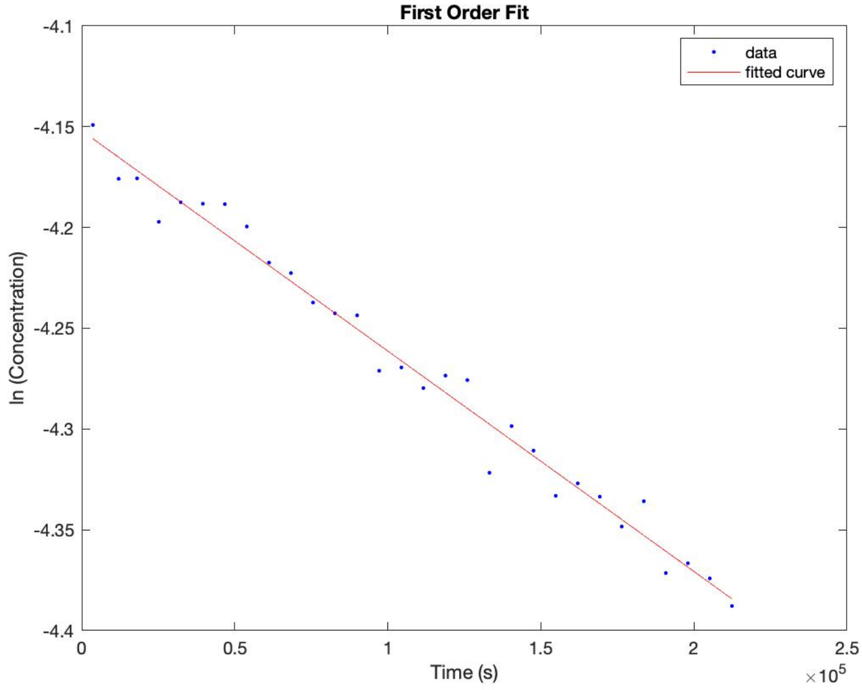

Fig. S3. Graph of ln[2NDP] *vs.* time for the un-catalysed (background) reaction shown in **Fig. S2(a)**: pD 8.32, 2NDP (15.8 mM) substrate, no cage catalyst. A least squares linear fit was used to determine the 1st order rate constant for the reaction.

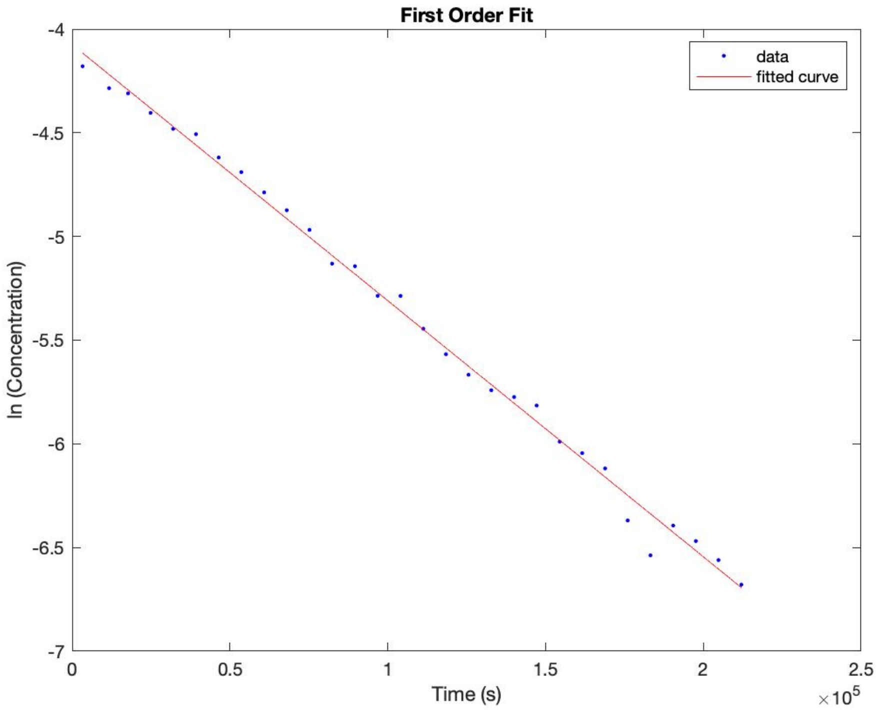

Fig. S4. Graph of ln[2NDP] *vs.* time for the cage-catalysed reaction shown in **Fig. S2 (b)**: pD 8.32, 2NDP (15.8 mM) substrate, **H** (0.627 mM) as catalyst. A least squares linear fit was used to determine the 1st order rate constant for the reaction.

In order to account for small variations in pD for experiments with and without cage **H** catalyst present, the background reaction rate of hydrolysis with each substrate was measured over a range of pD values. A plot of background rate constant *vs.* pD (**Fig. S5**) gave a linear relationship from which a calculated background reaction rate could be extracted for any pD value within the range tested, to be compared with the precise catalysed reaction rates. These calculated values (**Calc_K$_{bk}$**) can be found in **Table S2**.

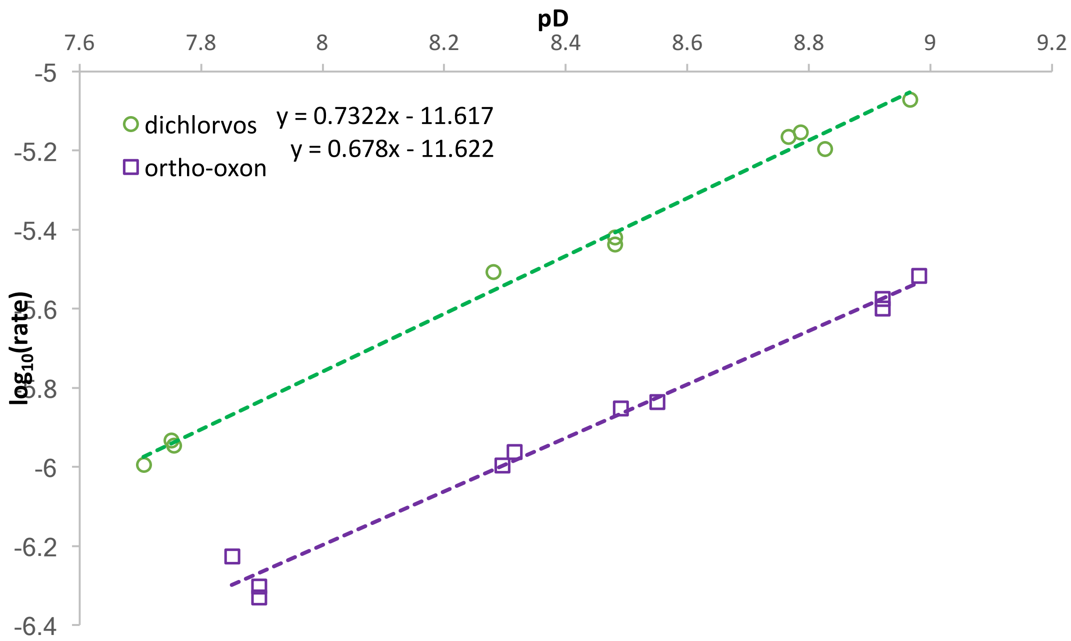

Fig. S5. Plot of measured background hydrolysis rates (first order), $\mathbf{K_{bk}}$, against pD for dichlorvos (green circles) and 2NDP (purple squares). The displayed equations (top left) from a linear fit to these data were used to obtain by interpolation an accurate background hydrolysis rate to match each catalysed reaction for any pD value in the range; see (**Calc_$\mathbf{K_{bk}}$**) values in **Table S2**.

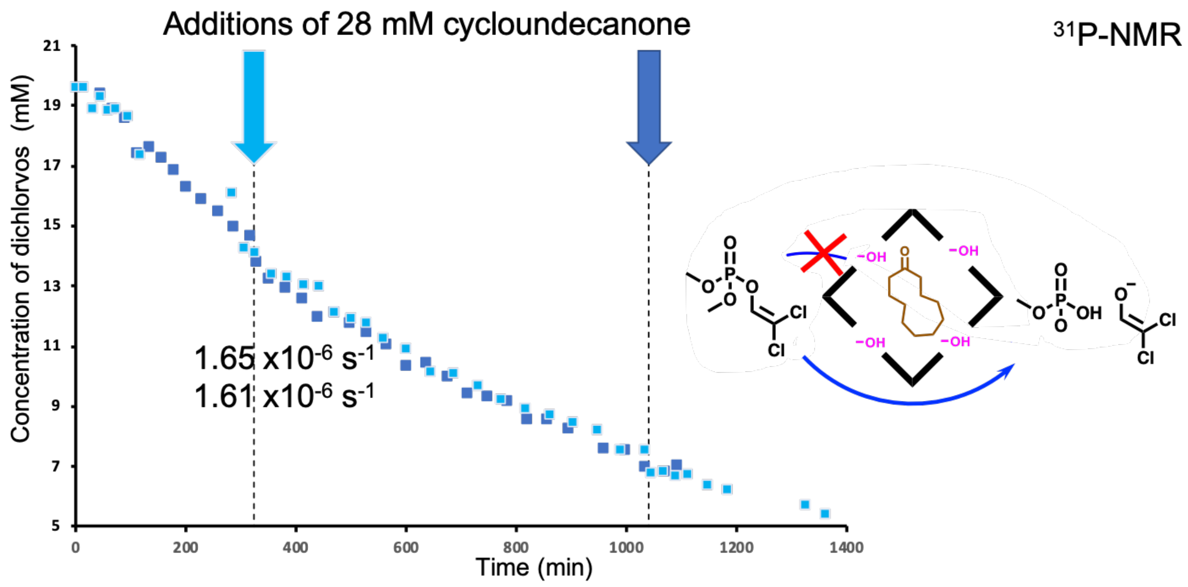

Fig. S6. Control Experiment 1: blocking the cage cavity of H with cycloundecanone. Cage catalysed hydrolysis of dichlorvos (measured by ^{31}P NMR) with addition of the strongly-binding guest cyclo-undecandone (CUD) at times highlighted by vertical line: run 1, dark blue; run 2, light blue. Conditions: 19.9 mM dichlorvos; 0.5 mM **H**, pD 8.15; 10 mM DMMP as ^{31}P NMR marker; 28 mM CUD added when indicated. See **Table 2** (lines *f, g*) for the numerical data. It is clear that addition of the CUD results in no significant change to the progress of the hydrolysis of dichlorvos.

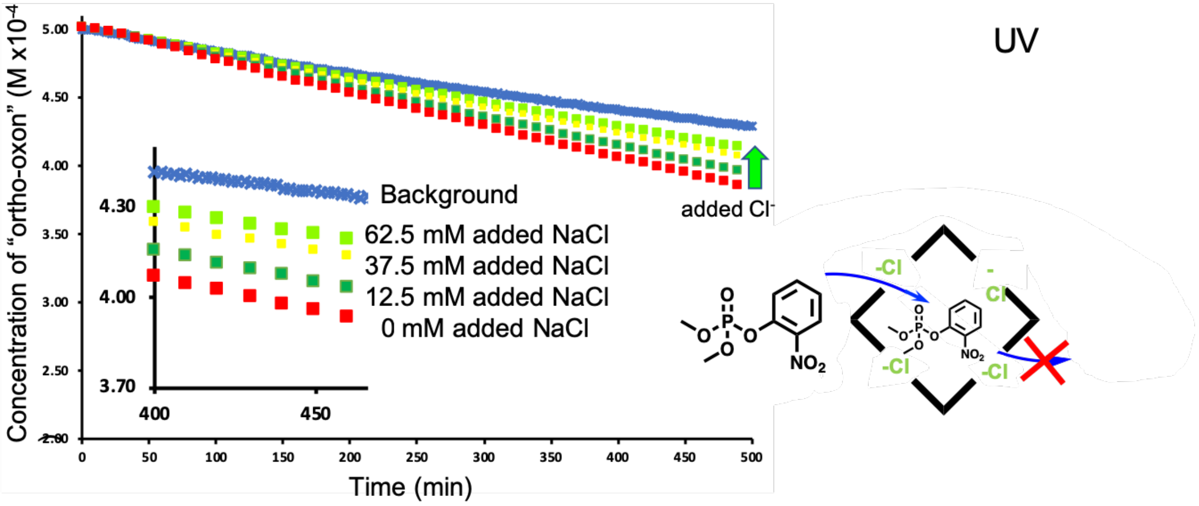

Fig. S7. Control experiment 2: inhibition of the catalysed reactions using excess chloride

Plot showing the consumption of starting material over time for catalysed hydrolysis of 2NDP by H showing the weakly inhibiting effect of added chloride. Conditions: 0.5 mM 2NDP; 0.21 mM **H**, pH 8.55; 303 K) with different additions of NaCl. Measurements were performed by monitoring the absorbance of the product 2-nitrophenolate at 420 nm. The results show a slow but incomplete decrease in reaction rate towards the background rate as more chloride is added. See **Table S3** (lines *m, n, o, p, q*).

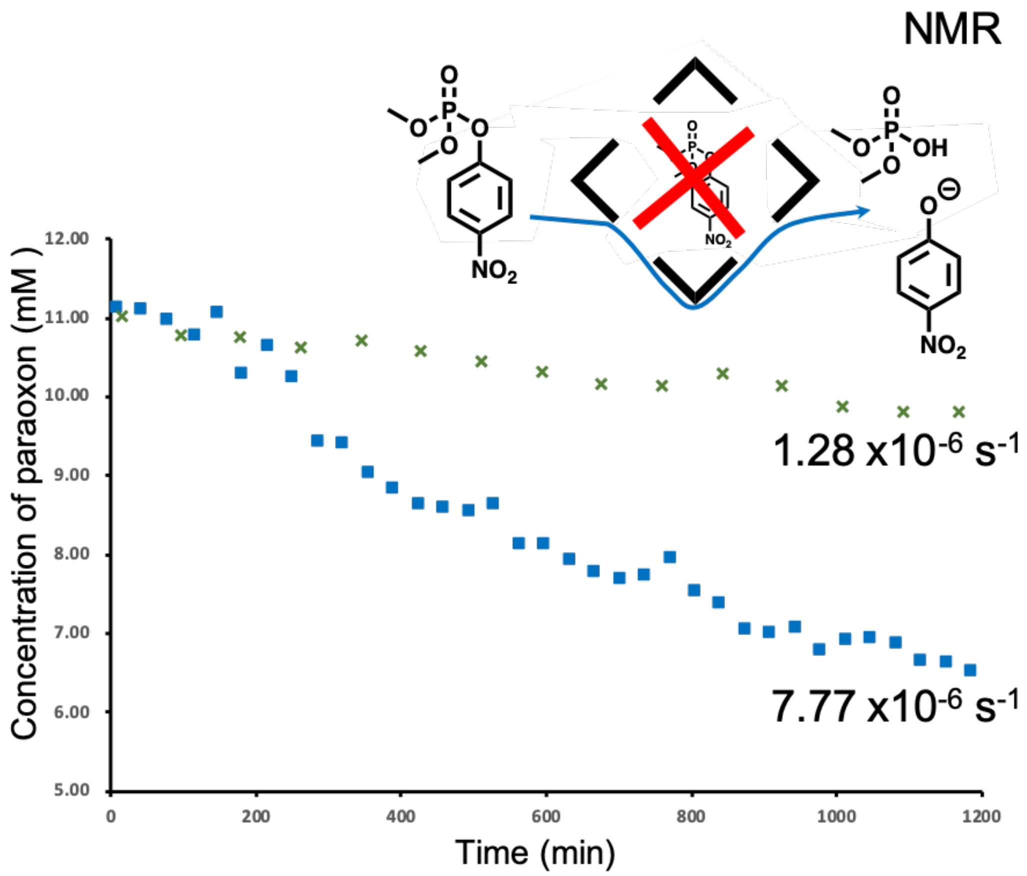

Fig. S8. Control experiment 3: catalysis with a substrate that does not bind (4NDP). Progress of hydrolysis of 4NDP with/without cage **H** catalyst, (17.5 mM 4DNP, 0.95 mM **H**, pD 8.55, 25 °C, followed by ^{31}P NMR spectroscopy). See **Table S2** (lines *k, h*) for the numerical data.

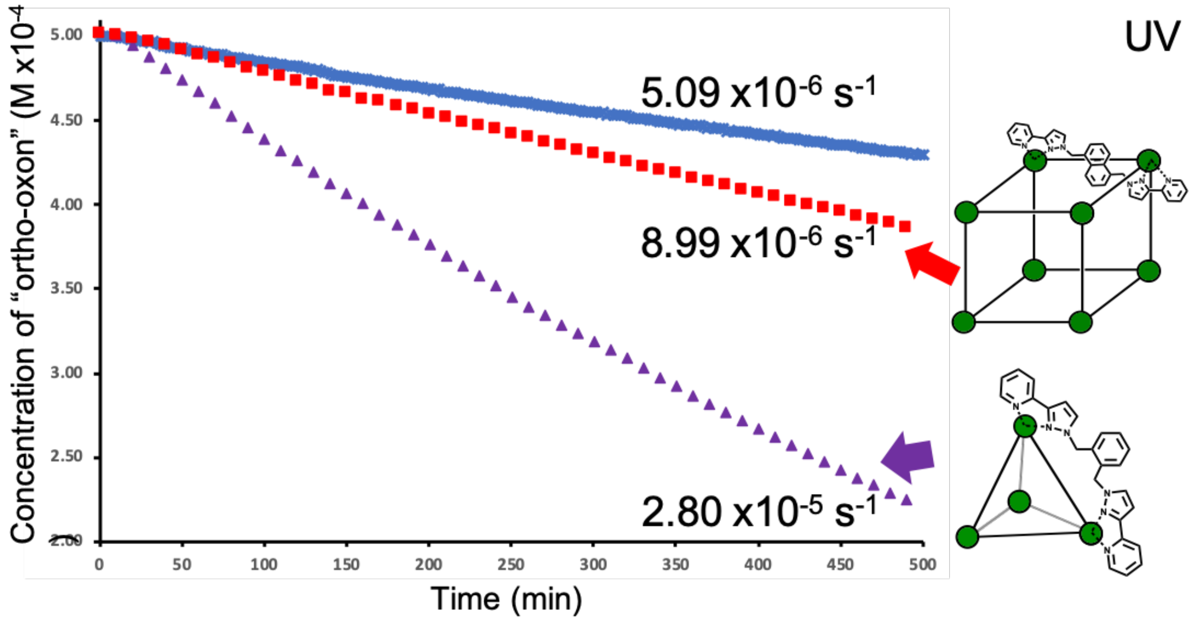

Fig. S9. Control experiment 4: catalysis with the non-binding cage T
Graph comparing the progress of reactions for hydrolysis of 2NDP without any type of cage catalyst (blue line), catalysed by 0.21 mM **H,** and catalysed by 0.4 mM **T** (purple), where the concentrations of the two different catalysts are adjusted to provide the same concentration of metal ion / ligand components**.** Substrate was 0.5 mM 2DNP, pH 8.55, 30 ^{o}C. See **Table S3** (entries *m, q, r*) for the numerical data.

Substrate Name	Substrate Conc (mM)	pH	K_{bk} (s^{-1})	Average K_{bk} (s^{-1})	RMSE	% error	
4NDP	1.0	8.55	2.985E-06	2.990E-06	0.0002	0.01%	
	0.8		2.984E-06		0.0002	0.01%	
	0.5		3.002E-06		0.0002	0.01%	
2NDP	1.0		4.993E-06	5.033E-06	0.0009	0.03%	
	0.8		5.012E-06		0.0009	0.03%	
	0.5		5.094E-06		0.0014	0.04%	r

K_2 = (Kobs-Kbk)/[Cage]

$$\% \text{ error} = -2 \times \frac{RMSE}{ln([Substrate])}$$

Substrate Name	Substrate Conc (mM)	pH	K_{obs} (s^{-1})	K_{obs}-K_{bk} (s^{-1})	Cage Name	Cage Conc (mM)	Inhibitor Conc [NaCl] (mM)	K_2 (M^{-1} s^{-1})	RMSE	% error	
2NDP	0.5	8.55	8.996E-06	3.963E-06	H	0.2065	0.0	1.919E-02	0.0016	0.04%	m
	0.4		9.250E-06	4.217E-06			0.0	2.042E-02	0.0016	0.04%	
	0.2		9.313E-06	4.280E-06			0.0	2.073E-02	0.0030	0.07%	
	0.5		8.074E-06	3.041E-06			12.5	1.473E-02	0.0013	0.03%	n
	0.4		8.315E-06	3.282E-06			12.5	8.205E-03	0.0014	0.04%	
	0.2		8.167E-06	3.134E-06			12.5	7.835E-03	0.0021	0.05%	
	0.5		7.108E-06	2.075E-06			37.5	5.189E-03	0.0010	0.03%	o
	0.4		7.552E-06	2.519E-06			37.5	6.298E-03	0.0012	0.03%	
	0.2		7.274E-06	2.241E-06			37.5	1.085E-02	0.0016	0.04%	
	0.5		6.583E-06	1.550E-06			62.5	7.507E-03	0.0006	0.02%	p
	0.4		7.010E-06	1.977E-06			62.5	9.574E-03	0.0009	0.02%	
	0.2		7.097E-06	2.064E-06			62.5	9.996E-03	0.0029	0.07%	
	0.5		2.766E-05	2.263E-05	T	0.4	0.0	5.657E-02	0.0103	0.27%	q
	0.4		2.858E-05	2.355E-05			0.0	5.886E-02	0.0102	0.26%	
	0.2		2.864E-05	2.361E-05			0.0	5.902E-02	0.0126	0.29%	
	0.5		2.766E-05	2.263E-05			12.5	5.657E-02	0.0093	0.24%	
	0.4		3.006E-05	2.503E-05			12.5	1.212E-01	0.0129	0.32%	
	0.2		2.809E-05	2.306E-05			12.5	1.117E-01	0.0119	0.27%	
	0.5		2.712E-05	2.209E-05			37.5	1.069E-01	0.0082	0.22%	
	0.4		2.828E-05	2.324E-05			37.5	1.126E-01	0.0099	0.25%	
	0.2		2.887E-05	2.384E-05			37.5	5.959E-02	0.0135	0.31%	
	0.5		2.625E-05	2.121E-05			62.5	5.304E-02	0.0078	0.21%	
	0.4		2.755E-05	2.252E-05			62.5	5.630E-02	0.0096	0.24%	
	0.2		2.802E-05	2.299E-05			62.5	5.746E-02	0.0101	0.23%	

Table S3 Data for the inhibition of catalysed hydrolysis of 2NDP in the presence of added chloride ions, determined by UV/Vis spectroscopy of the product (monitoring formation of 2-nitrophenolate at 420 nm). Left-hand tableL background reaction rates (no cage catalyst present). Right-hand table: cage-catalysed reaction rates. Highlighted data: *m, n, o, p, r* (**Fig. S7**); *m, q, r* (**Fig. S9**); *m, p* (main text, Table 1)

Substrate		pH	K_{obs}	K_{obs}-K_{bk}	Cage		K_2	RMSE	% error	
Name	Conc (mM)		(s^{-1})	(s^{-1})	Name	Conc (mM)	(M^{-1} s^{-1})			
4NDP	1.0	8.55	1.742E-05	1.443E-05	T	0.95	1.519E-02	9.000E-04	0.03%	s
	0.8		1.765E-05	1.466E-05			1.543E-02	1.100E-03	0.03%	
	0.5		1.794E-05	1.495E-05			1.574E-02	1.300E-03	0.03%	
	1.0		1.175E-05	8.759E-06		0.57	1.537E-02	6.000E-04	0.02%	
	0.8		1.186E-05	8.874E-06			1.557E-02	8.000E-04	0.02%	
	0.5		1.197E-05	8.984E-06			1.576E-02	9.000E-04	0.02%	
	1.0		7.306E-06	4.316E-06		0.285	1.514E-02	4.000E-04	0.01%	
	0.8		7.292E-06	4.302E-06			1.509E-02	5.000E-04	0.01%	
	0.5		7.539E-06	4.548E-06			1.596E-02	6.000E-04	0.02%	
2NDP	1.0		2.493E-05	1.990E-05		0.95	2.095E-02	7.100E-03	0.21%	t
	0.8		2.524E-05	2.021E-05			2.127E-02	6.900E-03	0.19%	
	0.5		2.590E-05	2.087E-05			2.197E-02	6.800E-03	0.18%	
	1.0		1.795E-05	1.292E-05		0.57	2.266E-02	2.900E-03	0.08%	
	0.8		1.785E-05	1.282E-05			2.249E-02	2.900E-03	0.08%	
	0.5		1.817E-05	1.313E-05			2.304E-02	3.100E-03	0.08%	
	1.0		1.174E-05	6.705E-06		0.285	2.352E-02	1.100E-03	0.03%	
	0.8		1.235E-05	7.316E-06			2.567E-02	1.300E-03	0.04%	
	0.5		1.163E-05	6.597E-06			2.315E-02	1.500E-03	0.04%	

Table S4. Data for hydrolysis of 2NDP and 4NDP in the presence of cage **T** determined by observing the appearance of the nitrophenolate product by UV/Vis absorption. Highlighted data: *s, t* (main text, **Table 1**).

Table S5: Single Crystal X-ray studies – crystallographic and data collection details

Compound	**H**•(dichlorvos)$_{1.56}$	**H**•DICP	**T**• (4-nitrophenolate)$_3$• (4NDP)$_2$
Empirical formula	$C_{375.16}H_{406.78}B_{16}Cl_{3.08}Co_8F_{64}N_{72}O_{39.16}P_{1.54}$	$C_{178.5}H_{177.5}B_4Cl_5Co_4F_{16}N_{36}O_{13}P_{0.5}$	$C_{202}H_{188}B_7ClCo_4F_{28}N_{41}O_{31.5}P_2$
Formula weight	8568.24	3810.75	4655.83
Temperature/K	150(1)	100(2)	100(1)
Crystal system	monoclinic	monoclinic	monoclinic
Space group	C2/c	C2/c	$P2_1/n$
a/Å	32.9035(9)	32.814(3)	21.31825(7)
b/Å	30.2026(9)	30.013(3)	24.92778(8)
c/Å	40.2361(11)	39.972(4)	35.69156(10)
β/°	95.761(3)	96.460(2)	90.7884(3)
Volume/Å^3	39783(2)	39116(6)	18965.30(7)
Z	4	8	4
ρ_{calc}g/cm^3	1.431	1.294	1.631
μ/mm^{-1}	3.693	0.486	0.456
F(000)	17690	15752	9524
Crystal size/mm^3	0.26 × 0.138 × 0.115	0.240 × 0.230 × 0.210	0.15 × 0.03 × 0.015
Radiation	CuKα (λ = 1.54184)	MoKα (λ = 0.71073)	Synchrotron (λ = 0.6889)
2Θ range for data collection/°	5.398 to 101.07	1.844 to 50.234	1.932 to 65.126
Index ranges	-32 ≤ h ≤ 32, -30 ≤ k ≤ 29, -39 ≤ l ≤ 40	-38 ≤ h ≤ 39, -35 ≤ k ≤ 34, -47 ≤ l ≤ 47	-33 ≤ h ≤ 31, -38 ≤ k ≤ 38, -55 ≤ l ≤ 55
Reflections collected	76231	124461	378004
Independent reflections	20639 [R_{int} = 0.0561, R_{sigma} = 0.0473]	34477 [R_{int} = 0.1385, R_{sigma} = 0.1612]	75612 [R_{int} = 0.0882, R_{sigma} = 0.0793]
Data/restraints/parameters	20639/7731/2219	34477/272/2266	75612/6157/2677
Goodness-of-fit on F^2	1.041	1.103	1.084
Final R indexes [I>=2σ (I)]	R_1 = 0.1050, wR_2 = 0.2909	R_1 = 0.1098, wR_2 = 0.3042	R_1 = 0.0713, wR_2 = 0.1973
Final R indexes [all data]	R_1 = 0.1224, wR_2 = 0.3047	R_1 = 0.2107, wR_2 = 0.3684	R_1 = 0.1172, wR_2 = 0.2235
Largest diff. peak/hole / e Å^{-3}	1.28/-0.89	2.19/-1.00	1.69/-0.92

Data were collected at (i) the University of Warwick on a Rigaku / Oxford Diffraction 'Supernova' diffractometer; (ii) the University of Sheffield on a Bruker Apex II diffractometer; or (iii) at the UK Diamond Light Source Synchrotron facility. Crystals of **H**•(dichlorvos)$_{1.56}$ and **H**•DICP were prepared using the 'crystalline sponge' method as described in the main text; crystals of **T**•(4-nitrophenolate)$_3$•(4NDP)$_2$ were grown by slow evaporation of an NMR sample of **T** in the presence of excess 4NDP in D_2O.

All structure determinations suffered from the usual weak scattering characteristic of crystals of this type, associated with large unit cells and disorder of solvents / anions. This necessitated extensive use of restraints during the refinements to achieve stable and chemically reasonable models. Some anions / solvents were refined with fractional occupancies over two sites when the disorder could be modelled. Large solvent-accessible voids containing diffuse electron density that could not be satisfactorily modelled were accounted for using the SQUEEZE command in PLATON. Full details of the treatments of the three structures, including software used, are given in the individual CIFs. **CCDC deposition numbers: 1959405-1959407.**

References

S1 I. S. Tidmarsh, T. B. Faust, H. Adams, L. P. Harding, L Russo, W. Clegg, M. D. Ward, *J. Am. Chem. Soc.* 2008, **130**, 15167.

S2 M. Whitehead, S. Turega, A. Stephenson, C. A. Hunter, M. D. Ward, *Chem. Sci.* 2013, **4**, 2744.

S3 C. G. P. Taylor, W. Cullen, O. M. Collier, M. D. Ward, *Chem. Eur. J.* 2017, **23**, 206.

S4 W. Cullen, S. Turega, C. A. Hunter, M. D. Ward, *Chem. Sci*. 2015, **6**, 2790

S5 C. G. P. Taylor, J. R. Piper, M. D. Ward, *Chem. Commun*. 2016, **52**, 6225.

S6 W. Cullen, A. J. Metherell, A. B. Wragg, C. G. P. Taylor, N. H. Williams, M. D. Ward, *J. Am. Chem. Soc.* 2018, **140**, 2821.

S7 J. S. Fleming, K. L. V. Mann, C.-A. Carraz, E. Psillakis, J. C. Jeffery, J. A. McCleverty, M. D. Ward, *Angew. Chem., Int. Ed.*, 1998, **37**, 1279.

S8 S. Turega, M. Whitehead, B. R. Hall, A. J. H. M. Meijer, C. A. Hunter, M. D. Ward, *Inorg. Chem*. 2013, **52**, 1122.
